# Outcome of CentriMag™ extracorporeal mechanical circulatory support use in critical cardiogenic shock (INTERMACS 1) patients

**DOI:** 10.1007/s12055-020-01060-6

**Published:** 2020-09-30

**Authors:** Vipin Mehta, Rajamiyer V. Venkateswaran

**Affiliations:** 1 Wythenshawe Hospital, Manchester University NHS Foundation Trust, Southmoor Road, Manchester, M23 9LT UK; 2 Faculty of Medicine and Biology, University of Manchester, Manchester, UK

**Keywords:** Critical cardiogenic shock, CentriMag™, Mechanical circulatory support, Bridge to decision, ECMO

## Abstract

**Purpose:**

Prognosis of patients presenting with INTERMACS 1 critical cardiogenic shock is generally poor. The aim of our study was to investigate the results of CentriMag™ extracorporeal short-term mechanical circulatory support as a bridge to decision in patients presenting with critical cardiogenic shock in our unit.

**Methods:**

We retrospectively analysed 63 consecutive patients from January 2005 to June 2017, who were treated with a CentriMag™ device at our institution as a bridge to decision. Patients requiring extracorporeal support for post-cardiotomy shock and for primary graft dysfunction after heart transplantation were excluded.

**Results:**

Patients’ median age was 44 years (IQR 31–52, range 15.4–62.0) and 42 (67%) were male. Primary diagnosis at presentation was ischaemic cardiomyopathy (*n* = 24; 38.1%), viral myocarditis (*n* = 19; 30.2%), idiopathic dilated cardiomyopathy (*n* = 8; 12.7%), and others (*n* = 12; 19%). The median duration of support was 25 (IQR 9.5–56) days. A total of 7 (11%) patients were supported with peripheral veno-arterial (VA) extra corporeal membrane oxygenation (ECMO), 6 (9%) with central VA ECMO, 8 (13%) with left ventricular assist device (LVAD), 17 (27%) with biventricular assist device (BiVAD), and 25 (40%) with ECMO and then converted to BiVAD. Overall, 22 (34.9%) patients died while on CentriMag™ mechanical circulatory support. Complications included bleeding requiring reoperation/intervention in 24 (38%), renal failure requiring dialysis in 29 (46%), bacterial infections in 23 (37%), fungal infections in 15 (24%), critical limb ischaemia in 6 (10%), and stroke in 8 (13%). The overall survival to successful explant from CentriMag™ was 65.1% (*n* = 41) and survival to hospital discharge was 58.7% (*n* = 37). Of these, 10 (16%) had cardiac recovery and were successfully explanted, 20 (32%) were bridged to heart transplantation, 11 (17%) were bridged to long-term left ventricular assist device, 3 (4.7%) were later on transplanted, and 1 (1.6%) recovered to decommissioning. The 1-, 5-, and 10-year survival rates were 55%, 46%, and 23% respectively.

**Conclusion:**

Our results demonstrate an excellent outcome with the use of the CentriMag™ device in this seriously ill population. Despite requiring multiple procedures, over 58% of patients were discharged from hospital with 5-year survival of 46%.

## Introduction

Refractory heart failure despite maximal medical therapy carries a poor prognosis. These patients often present acutely with no time for full assessment for heart transplantation. In many of them, even the cause of heart failure is unclear at presentation. These patients have been supported with a variety of mechanical options, including intra-aortic balloon pump (IABP), percutaneous devices like Impella®, extra corporeal life support (ECLS) with CentriMag™, and implantation of durable devices like HeartMate 3™.

These mechanical device strategies allow time to stabilise the haemodynamics in these very sick patients, to perform the diagnosis of the cause of heart failure, and to allow other secondarily failing organs to recover.

The UK national annual report [1] on mechanical circulatory support for 2018–2019 shows that during 2018/2019, there were 90 short-term device implantations into 68 patients, with 15% patients explanted at 30 days due to myocardial recovery. The 30-day mortality was 30% and 1-year survival in UK was 38.9% (including those transplanted). This strategy as bridge to decision is deemed cost effective and therefore funded via the National Health Service (NHS) in UK.

There is a lack of consensus on the best modality for circulatory support in critical cardiogenic shock (INTERMACS 1). The practice and results vary based on local expertise and resources. We are the biggest centre in UK for short-term mechanical support [1] and have therefore developed expertise in looking after these patients at our centre with much better results than the UK national results, using CentriMag™ as the primary support device. We retrospectively analysed our practice over the last 12 years to evaluate the outcomes and complications of our device strategy.

## Materials and methods

We retrospectively analysed 63 consecutive patients from January 2005 to June 2017, who were treated with a CentriMag™ device (Abbott Laboratories, Abbott Park, IL) at our institution as a ‘bridge to decision’ which means these patients had multi-organ dysfunction at presentation due to low cardiac output and therefore were not suitable directly for transplantation/durable LVAD.

The aim of our study was to analyse the outcomes of patients supported with short-term extracorporeal mechanical circulatory support presenting in critical cardiogenic shock (INTERMACS class 1). The primary end point of the study was survival to discharge from hospital, long-term survival at 1 year and 5 years. The secondary end point of the study was to analyse the associated complications, duration of intensive treatment unit (ITU) stay, and hospital stay.

### Inclusion criteria

Inclusion criteria include all patients who presented acutely at our institution, with critical cardiogenic shock, and underwent mechanical circulatory support with a short-term extracorporeal device.

### Exclusion criteria

We excluded the following patients:

1. patients who directly underwent implantation of durable LVAD like HeartWare/HeartMate 2 or 3;
2. patients who required planned or urgent extracorporeal support after routine cardiac surgery; and
3. post-transplant patients who required ECMO/circulatory support for primary graft dysfunction after heart or lung transplantation.

### Data collection

Data was collected from prospectively kept databases and case notes. Intensive care data and complications were recorded from the intensive care electronic patient record. The patient length of stay in hospital and intensive care were recorded from the hospital’s patient administration system. The device strategy was recorded from the operation notes and long survival was recorded from the hospital database which is linked to the national register for birth and deaths.

### Statistical analysis

Data was analysed using Microsoft Excel and R and SPSS software. Descriptive data are presented as mean with range and median with interquartile range, along with percentages. Survival curve and numbers at risk were generated using the Kaplan-Meier survival analysis, which is a non-parametric statistic used to estimate the survival function. Groupwise comparison in Table 1 was intentionally not done as these are self-selected groups based on outcome and therefore not directly comparable.

**Table 1:** Characteristics of 4 self-selected groups based on outcome

	Recovered (*n* = 10)	Durable LVAD (*n* = 11)	Transplanted (*n* = 20)	Died (*n* = 22)	Total (*n* = 63)
Age, in years (mean ± SD)	39 ± 10	37 ± 15	41 ± 13	44 ± 14	41 ± 14
Sex (male:female)	4:6	7:4	19:1	12:10	42:21
Duration of support (in days, mean ± SD)	26 ± 23	42 ± 29	45 ± 36	27 ± 28	35 ± 31
Total ITU stay (in days, mean ± SD)	31 ± 23	69 ± 37	57 ± 35	26 ± 20	48 ± 35
Total in-hospital stay (in days, mean ± SD)	37 ± 23	95 ± 61	77 ± 39	27 ± 19	63 ± 47
Complications					
Bleeding	3/10	5/11	8/20	8/22	24 (38%)
Bacterial infection	3/10	6/11	9/20	5/22	23 (37%)
Fungal infection	6/10	1/11	5/20	3/22	15 (24%)
Stroke	0/10	2/11	1/20	5/22	8 (13%)
Critical limb ischaemia	1/10	1/11	0/20	4/22	6 (10%)
Renal failure requiring haemofiltration	5/10	4/11	11/20	9/22	29 (46%)

Statistical *p* values not calculated as groups are not directly comparable

## ECMO technique, early conversion to BiVAD, and management principles

**Fig. 1:**
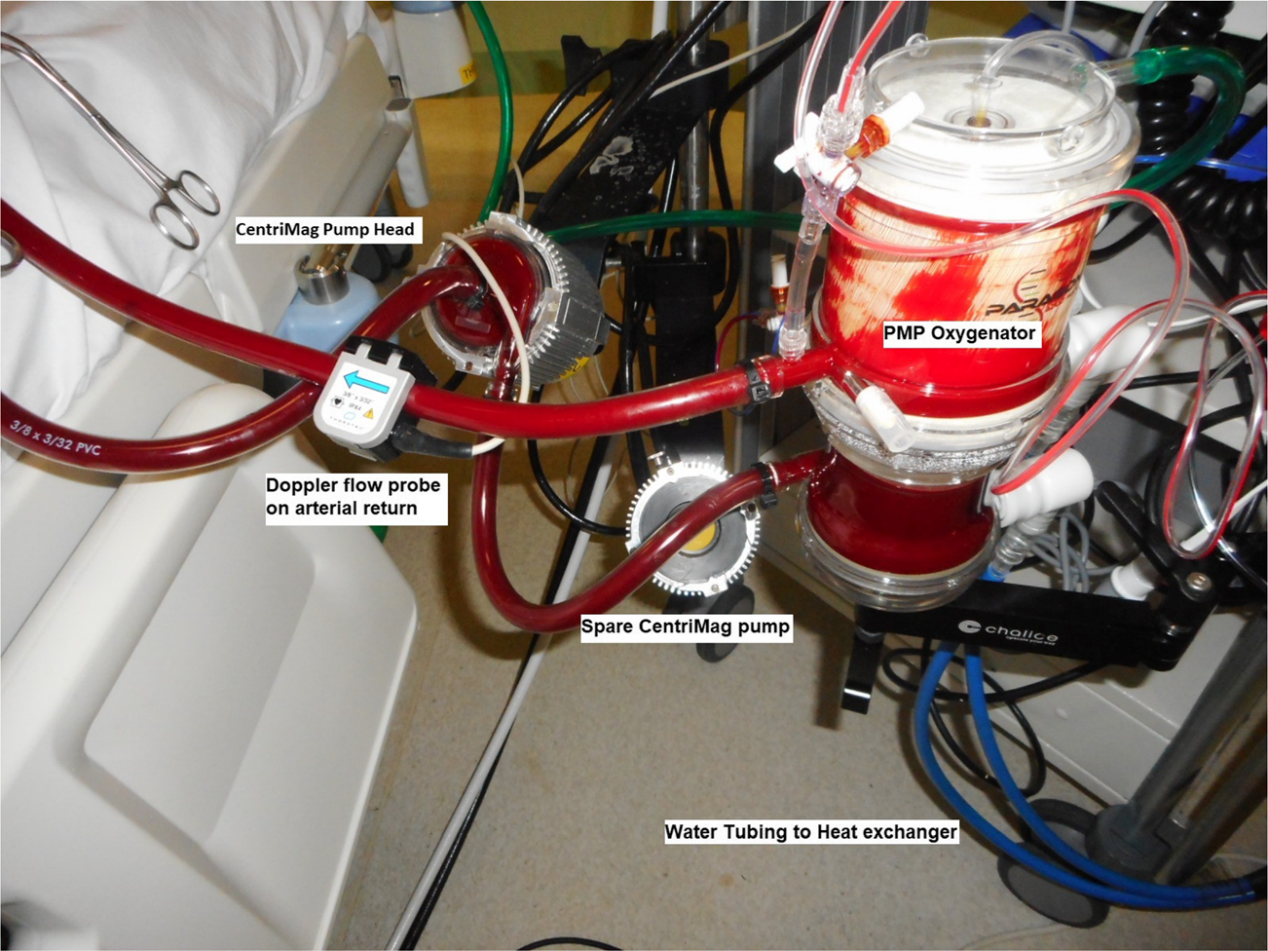
ECMO circuit showing CentriMag™ as bridge to decision for circulatory support in critical cardiogenic shock

We utilised either a central or a peripheral cannulation technique to deliver veno-arterial (VA) ECMO. In the central ECMO technique, the aortic cannula (Medtronic EOPA®—Elongated One-Piece Arterial) is tunnelled under the sternum and brought out below the costal margin. The venous cannula is tunnelled under the xiphisternum on the right side using a malleable (Medtronic) size 32-mm cannula inserted into the right atrium. In the peripheral ECMO technique, a right axillary artery with 8-mm polytetrafluoroethylene (PTFE) side graft is used for arterial return and a heparin-coated wire-reinforced long-term femoral venous pipe (Maquet® HLS cannula with Bioline coating) is used for drainage. The ECMO circuit consists of a magnetically levitated centrifugal pump (Abbott® CentriMag™) and a polymethylpentene oxygenator (Paragon™) as shown in Fig. 1.

The heart is completely rested as soon as the ECMO support is commenced. Most of the inotropic support is weaned and only vasoconstrictors/IABP support is maintained. The lungs are ventilated according to usual critical care practice, and the ECMO pump speed is adjusted to maintain some ventricular ejection. This is important in order to maintain flow through the pulmonary vasculature to prevent stasis and reduce the risk of pulmonary venous and intra-cardiac thrombus formation. End-tidal CO_2_ levels and Swan-Ganz catheter–transduced ejection trace are used to monitor pulmonary blood flow. Heparinisation is reversed with protamine and, after thorough haemostasis, the chest is closed.

In the immediate postoperative period, activated clotting time (ACT) levels are checked every 4–6 h and heparin is usually not started until bleeding risk is considered minimal, and ACT level falls below 180 s. Heart function is monitored daily in the postoperative period and if there is an improvement, then ECMO support is gradually weaned and inotropic support increased. Echocardiographic evidence of ventricular recovery, together with an improved arterial pressure trace and stable blood pressure with a flow of under 2.8 L/min, is our principal indication for a trial of ECMO weaning.

If the heart does not recover function, then within 1 week, ECMO is converted to central biventricular assist device (BiVAD) with an oxygenator in the left side circuit.

We always use median sternotomy for these patients to convert to BiVAD. For right ventricular assist device (RVAD) circuit, the venous cannula is tunnelled under the xiphisternum on the right side using a malleable (Medtronic) size 32-mm cannula inserted into the right atrium. The outflow of RVAD is again tunnelled under the xiphisternum and using Medtronic EOPA® (Elongated One-Piece Arterial cannula) into the pulmonary artery. We used an 8–10-mm vascular graft anastomosed end to side to the pulmonary artery and pass the EOPA® cannula through it, which tends to reduce bleeding and makes explant easier. Similarly, for LVAD circuit, we use a wire-reinforced CentriMag™ 34-Fr drainage (venous) cannula kit, tunnelled under the left costal margin to cannulate the left ventricle (LV) apex and arterial return uses EOPA® cannula tunnelled under the xiphisternum, and passed into the ascending aorta via an 8–10-mm vascular graft. Both circuits consist of a magnetically levitated centrifugal pump (Abbott® CentriMag™) and a polymethylpentene (PMP) oxygenator (Paragon™) is added to the LVAD circuit.

**Fig. 2:**
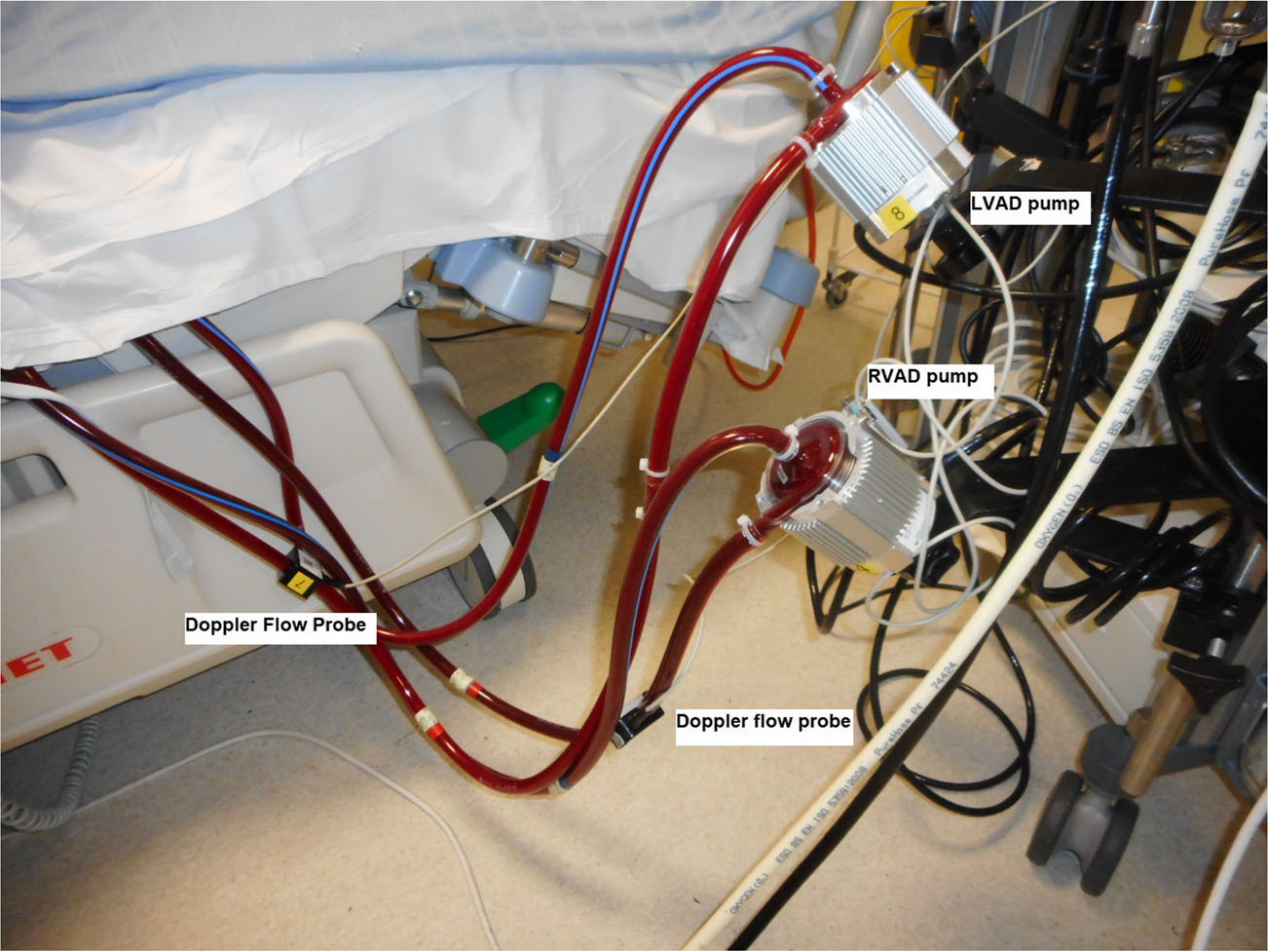
BiVAD circuit showing CentriMag™ for circulatory support in critical cardiogenic shock

The recovery of lung function is assessed by lung compliance measured on a ventilator, end-tidal CO_2_ trace, and serial chest X-rays. Once the lungs have recovered, the oxygenator is removed, which helps in reducing the consumptive coagulopathy and improves platelet counts. CentriMag™ pumps in BiVAD configuration are shown in Fig. 2.

IABP is removed and a thorough assessment is done for potential candidacy for heart transplantation. This assessment involves complete medical history to exclude any drug abuse, compliance and psycho-social issues, HLA typing and testing for pre-formed antibodies, and whole-body computed tomography (CT) scan to rule out stroke and cancer.

**Fig. 3:**
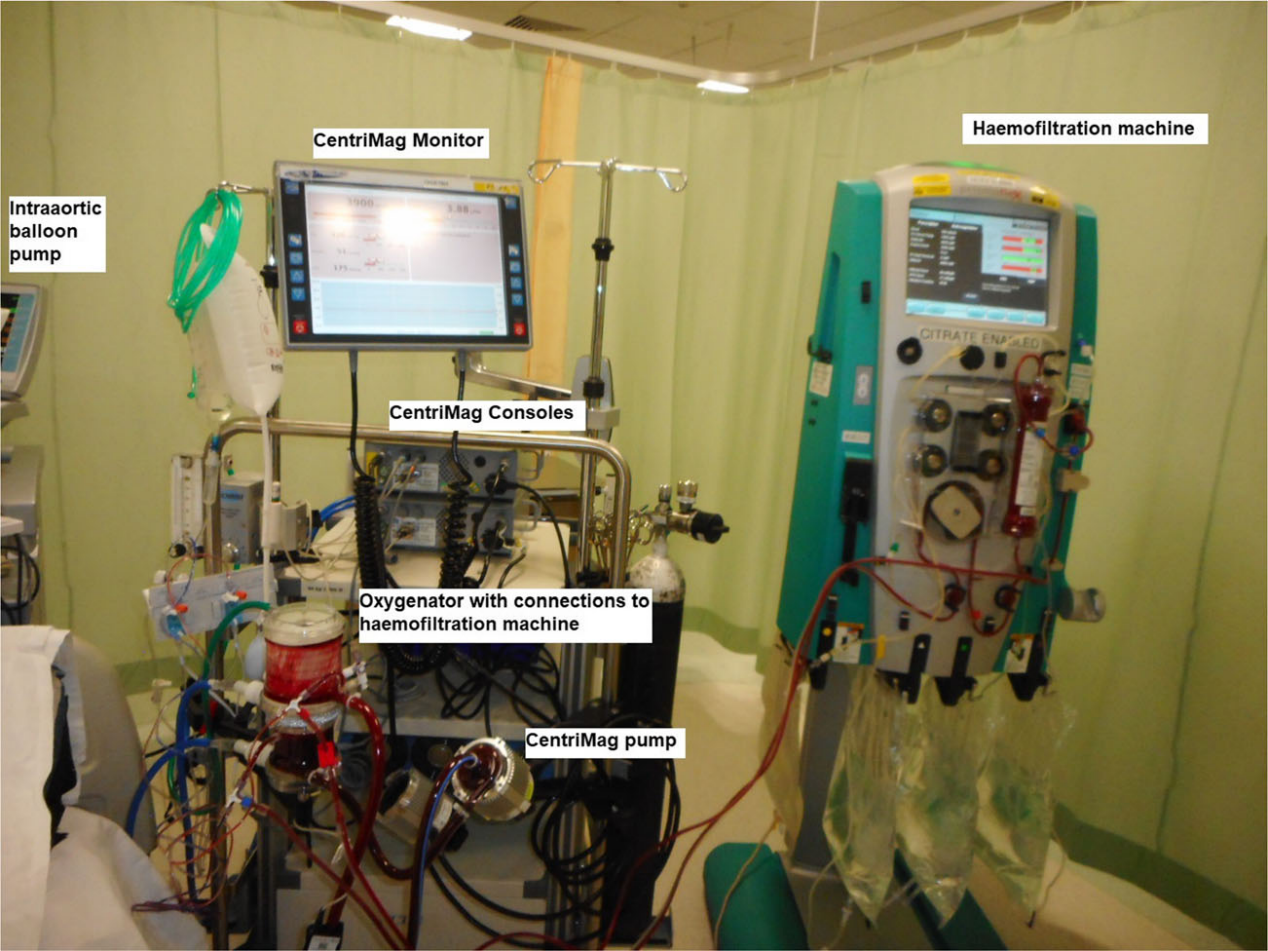
Circuit with haemofiltration machine connections, showing CentriMag™ pump and console with monitor

These patients frequently need renal support with haemofiltration, tracheostomy, nasogastric (NG) feeding, close monitoring for infection and antibiotics, and weekly line changes. These patients frequently develop venous thrombosis due to multiple vascular access for ventral lines and dialysis catheters. In such patients, to reduce the vascular access complications, a haemofiltration circuit can be connected to the ECMO/BiVAD circuit as shown in Fig. 3.

The patients are given intensive physiotherapy in bed and mobilised into chair and then gradually they are supported to walk in the corridor, all while attached to the BiVAD. The aim for this period of treatment is to convert multiple organ failure into single-organ dysfunction and provide good cardiac output to allow kidneys, liver, lungs, brain, and muscle strength to recover. The CentriMag pump head is licenced for use up to 30 days. If any thrombosis is suspected or patients stay longer on short-term circulatory support, then the pump head is changed every 30 days. They remain on heparin anticoagulation while on BiVAD as frequent line changes, tracheostomy, gastrointestinal (GI) bleeding, frequent antibiotic changes, etc. can make warfarin therapy difficult.

## Decision-making for exit strategy

When all non-cardiac organs have fully recovered and patient is extubated and mobilising in ITU, at this stage, if heart has still not recovered, a decision is made for either listing for super-urgent heart transplantation or conversion to long-term durable LVAD support with implantable pumps like HeartWare or HeartMate 2/3. This decision is made at a multi-disciplinary team meeting consisting of transplant surgeons, transplant physicians, intensive care specialists, psychologists, social service teams, dieticians, transplant/ventricular assist device (VAD) specialist nurses, and physiotherapists. To assess suitability for durable LVAD, assessment of right ventricle (RV) function remains very difficult as right heart catheter study is not feasible on BiVAD. Surrogate markers are often used. If the patient is in ventricular fibrillation on BiVAD, they are not a candidate for durable LVAD. Turning down the RVAD pump speed can indicate reasonable RV function if central venous pressure (CVP) remains low and LVAD flow remains maintained. If they do not have pre-formed antibodies and have a common blood group/height and weight which means they will not wait a prolonged time awaiting a donor heart, then they usually go for primary heart transplant. If they have prolonged waiting times for donor organ due to uncommon blood group/height/weight, then they are considered for conversion to durable LVAD as a bridge to eventual heart transplantation. Destination LVAD implantation is currently not funded in UK.

## Results

### Demographics and baseline characteristics

Among the 63 consecutive patients from January 2005 to June 2017, their median age was 44 years (IQR 31–52) and their mean age was 40.9 years (min 15.4 years, max 62.0 years). Of these 63 patients, 42 (67%) were male.

Collectively, patients were supported for a total of 2202 days with median duration of support of 25 days (IQR 9.5–56 days), and mean duration of support was 35 days (min 2 days, max 124 days).

Primary diagnosis at presentation was ischaemic cardiomyopathy (*n* = 24; 38.1%), viral myocarditis (*n* = 19; 30.2%), idiopathic dilated cardiomyopathy (*n* = 8; 12.7%), specific (anthracycline/alcoholic/postpartum/Takotsubo) myopathy (*n* = 8; 12.7%), valvular heart disease (*n* = 3; 4.8%), and sarcoidosis (*n* = 1; 1.6%).

**Fig. 4:**
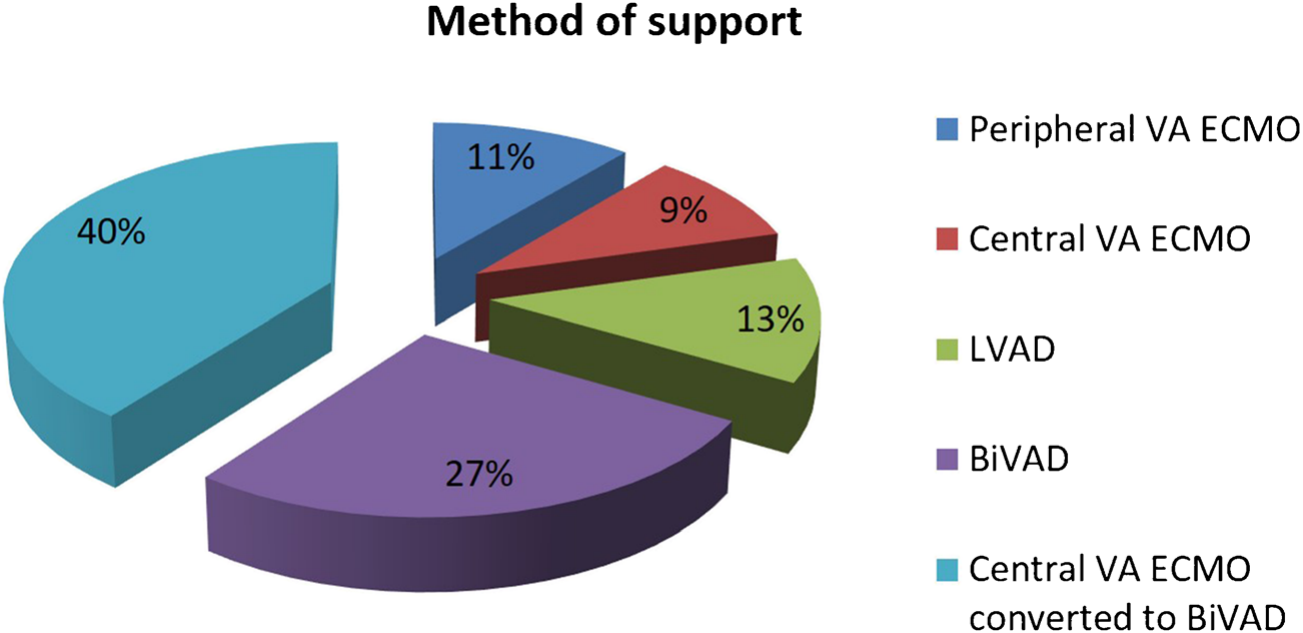
Different configurations of circulatory support in critical cardiogenic shock (INTERMACS 1) with CentriMag™ as bridge to decision

A total of 7 (11%) patients were supported with peripheral VA ECMO, 6 (9%) with central VA ECMO, 8 (13%) with LVAD, 17 (27%) with BiVAD, and 25 (40%) initially treated with central VA ECMO and then converted to short-term BiVAD as shown in Fig. 4.

### Primary outcomes

**Fig. 5:**
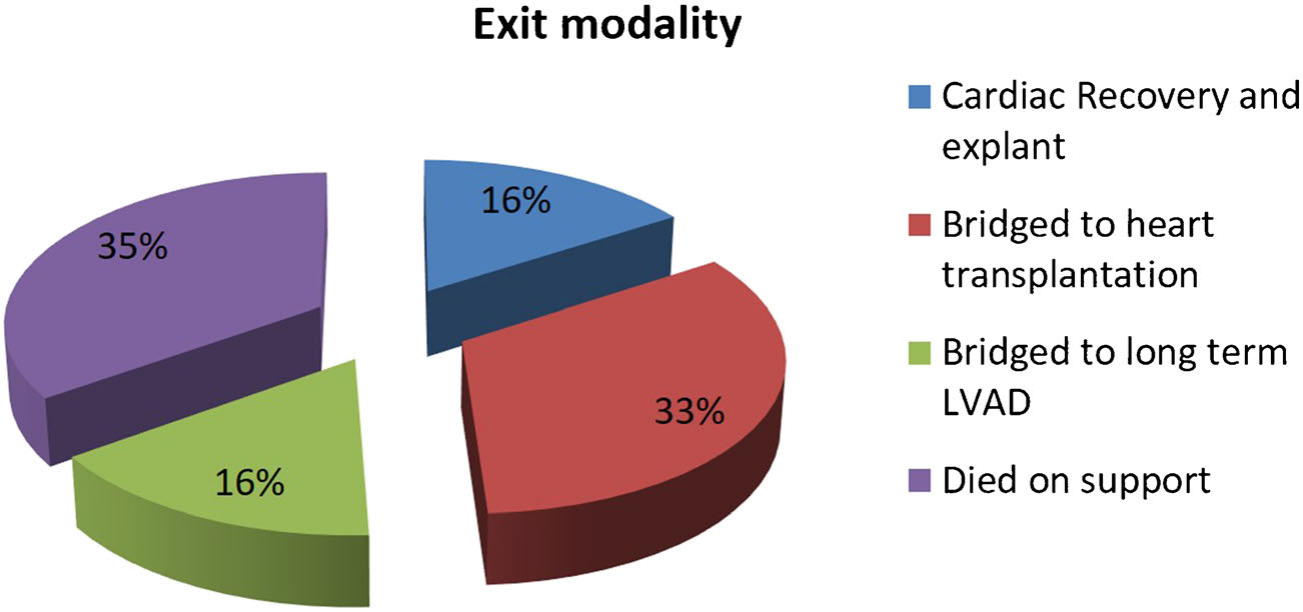
Exit strategy in patients on circulatory support in critical cardiogenic shock (INTERMACS 1) with CentriMag™ as bridge to decision

The survival to successful explant from CentriMag™ was 65.1% (*n* = 41) and survival to hospital discharge was 58.7% (*n* = 37). Of these, 10 (16%) had cardiac recovery and were successfully explanted, 20 (32%) were bridged to heart transplantation, 11 (17%) were bridged to long-term/durable left ventricular assist device, 3 (4.7%) were later on transplanted, and 1 (1.6%) recovered to decommissioning, as shown in Fig. 5.

Our 30-day survival rate was 71%; 90-day survival rate was 62%; while 1-, 5-, and 10-year survival rates were 55%, 46%, and 23% respectively.

**Fig. 6:**
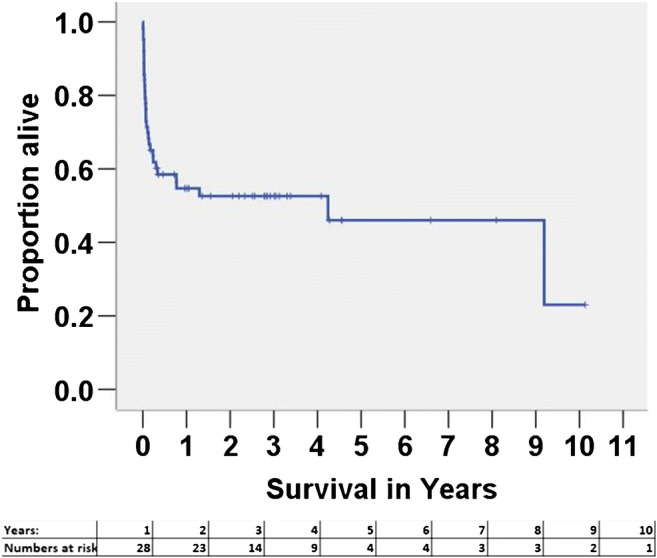
Survival curve for patients in critical cardiogenic shock (INTERMACS 1), supported with CentriMag™ as bridge to decision

**Fig. 7:**
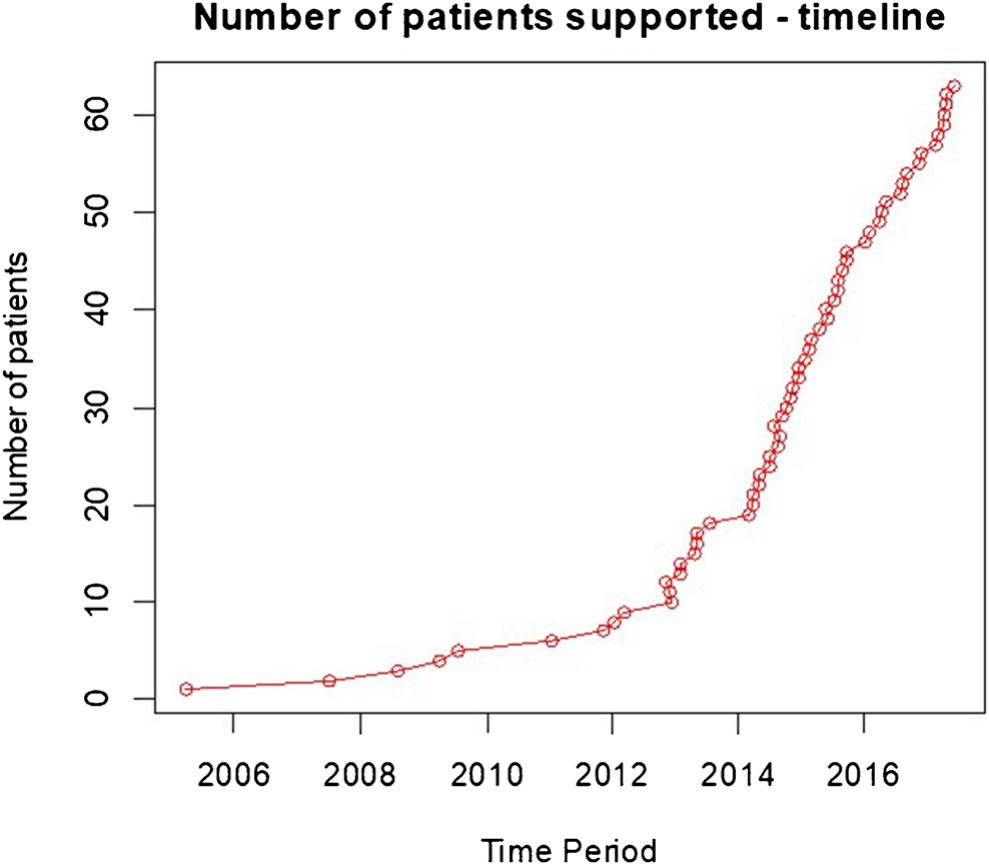
Timeline for patients supported in critical cardiogenic shock (INTERMACS 1) with CentriMag™ as bridge to decision

Kaplan-Meier survival curve is shown in Fig. 6 and timeline of patients is supported in Fig. 7.

### Secondary outcomes

Although few patients stayed much longer, the median ITU stay was 37 days (IQR 24–66 days) and the median in-hospital stay was 53 days (IQR 28–82 days) respectively. Mean ± standard deviation values are given in Table 1.

None of these patients was previously known/diagnosed with heart failure and their first presentation to our institution was in critical cardiogenic shock. Frequent diagnoses in the cardiac recovery group were viral/postpartum/Takotsubo cardiomyopathy/valvular heart disease. All these patients who had cardiac recovery and were successfully explanted from CentriMag support have had 100% survival rate so far, with none requiring transplantation/durable LVAD during follow-up.

Patients transplanted after short-term CentriMag circulatory support had an excellent long-term outcome with 5-year survival rate of 85% in our series. Those patients are by definition much sicker and their survival should not be compared with patients undergoing routine planned heart transplantation.

**Fig. 8:**
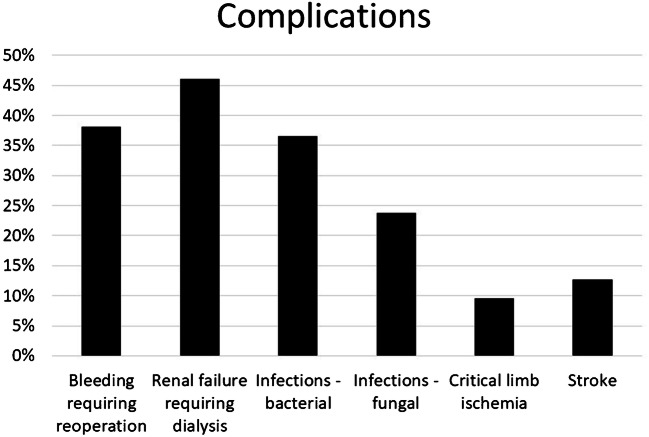
Complications in patients on circulatory support in critical cardiogenic shock (INTERMACS 1) with CentriMag™ as bridge to decision

Overall, 22 (34.9%) patients died while on CentriMag™ mechanical circulatory support. Complications included bleeding requiring reoperation/intervention in 24 (38%), renal failure requiring dialysis in 29 (46%), bacterial infections in 23 (37%), fungal infections in 15 (24%), critical limb ischaemia in 6 (10%), and stroke in 8 (13%), as shown in Fig. 8.

Excluding those who died (*n* = 22), there are three groups of survivors (recovery, durable LVAD, and transplanted). These groups are not directly comparable with each other as each is a self-selected group based on outcome. Their relative characteristics are given in Table 1.

## Discussion

The UK national annual report [1] on mechanical circulatory support for 2018–2019 shows that during 2018/2019, there were 90 short-term device implantations into 68 patients, comprising 50 VADs and 40 ECMO procedures. In UK, the majority (71%) of implantations were into INTERMACS profile 1 patients (critical cardiogenic shock). The median duration on short-term support was 11 days [1]. At 30 days post-implant, 25% of patients remained on short-term support, 12% had been transplanted, 17% were transferred to a long-term device, and 15% were explanted without transplant [1]. Thirty percent had died on support. Over the last decade, we are the biggest centre in UK for short-term mechanical support [1] and have therefore developed expertise in looking after these patients at our centre with much better results than the UK national results. Our 1-year survival rate is now 55%, while the national 1-year patient survival rate is 39%, from the point of the first short-term VAD implant (excluding those bridged to long-term support and not censored for transplant/explant) [1].

## Choice of device

Different centres have used various devices to support INTERMACS 1 patients. ECMO is the fastest way to stabilise a patient in acute cardiogenic shock and prevent end-organ failure, but it should likely be used for a short time and does not reduce the work of (‘unload’) the left ventricle. An intra-aortic balloon pump may provide improved diastolic perfusion of heart and other organs in a patient on ECMO. The TandemHeart provides significant support, but its insertion requires puncture of the atrial septum. The Impella fully unloads the left ventricle, critically reducing the work of the heart. Options for right ventricular support include the ECMO Rotaflow circuit, CentriMag, and Impella RP. The CentriMag is the most versatile device, allowing right, left, or biventricular support, but placement usually requires sternotomy.

### Role of Impella

Impella is a percutaneously inserted pump and can avoid need for sternotomy but the results are inferior to CentriMag when used for supporting INTERMACS 1 patients. Impella requires careful positioning across the aortic valve and frequently does not provide full circulatory support. This may explain inferior survival outcomes in studies using Impella. In a recent study by Tongers et al. [2], using Impella with VA ECMO showed 30-day survival rate of 49% and 6-month survival rate of 40%. Another study from Berlin [3] used Impella for left-sided mechanical circulatory support (MCS) and showed 37% in-hospital survival rate.

### Cardiohelp versus other portable ECMO devices

A review of Cardiohelp vs. other portable ECMO devices [4] showed that Cardiohelp had safer technological features, but on the other hand, was more complex to use. Considering the effectiveness, Cardiohelp was not statistically different from other technologies. According to the measures of safety and effectiveness, ECMO with Cardiohelp was not considerably different from other evaluated technologies [4]. Moreover, ECMO with Cardiohelp or CentriMag can be considered cost effective, provided that the patients are selected carefully in terms of neurological outcomes [4].

### Our choice: CentriMag™ with full MagLev™ technology

There are multiple pump devices available for short-term mechanical circulatory support. The common disadvantages of most centrifugal pumps include haemolysis and the fixed point of the ball-bearing for the propeller, which leads to stasis and pump thrombosis.

CentriMag™ with full MagLev™ technology (Abbott Laboratories, Abbott Park, IL, USA) is the device of choice at our institution due to its safety profile of low device-related thrombosis (2.5%) [5, 6], low haemolysis (5%) [5], and improved end-organ function [6]. These advantages are due to the following:

- Reduced blood trauma—a free-floating magnetically levitated, contact-free rotor prevents surface-to-surface contact that could cause blood trauma
- Minimised turbulence—wide blood flow pathways and absence of seals, bearings, and valves minimise blood turbulence and stasis
- Maintained stability—Automatic rotor positioning adjusts 50,000 times per second to maintain stable and consistent blood flow.

The CentriMag™ circulatory support system produces flows up to 9.9 litres per minute (LPM) with fewer rotations per minute [7] to help minimise blood trauma while still providing optimal flow support. The CentriMag™ system is fully transportable via air or ground ambulance using the compact system transporter, fits seamlessly into custom circuit designs to easily adapt to clinician preferences, and can be used with a variety of cannulation options.

### Comparison of outcomes

A retrospective study from Columbia University Medical Center, New York [8], 8], of 161 patients who received a CentriMag ventricular assist system between January 2007 and June 2014 found that device-related adverse events included major bleeding, infection, and stroke incidents occurring during CentriMag support. 

One hundred forty-three (88.8%) patients had biventricular VAD and 18 (11.2%) had isolated left VAD. The median duration of support was 16 days (interquartile range [IQR]: 10–29). Mortality was 24.8% and 1-year overall survival rate was 51.8% (95% CI: 43.3–59.5%) [8]. This is similar to our results with 1-year survival rate of 55%. Their most common adverse event during support was major bleeding (*n* = 121, 75.1%). Ninety-five (59.0%) patients developed major infections such as pneumonia and urinary tract infection [8], and sixteen (10%) patients experienced stroke [8]. Again, we had a similar range of complications in our study while direct comparison is difficult due to varying definitions of complications. They showed that stroke and reoperation caused by bleeding were rare beyond 30 days, whereas infection and non-surgical bleeding events were directly related to support time. They concluded that temporary VAD with CentriMag support is an effective treatment for patients in refractory cardiogenic shock [8] and despite its side effect profile including a high rate of blood transfusion early in the immediate postoperative period of CentriMag support, aggressive use of the CentriMag support device has acceptable survival to discharge and 1-year survival rate [8].

Another study using CentriMag support for INTERMACS 1 patients [9] showed 30-day post-implant survival was 79% (22 patients), which is similar to our 30-day survival of 71%. In this study, much higher proportion of patients were transplanted. Eighteen (64%) patients underwent transplantation, and 17 of them were discharged [9]. The mean support time was 40 days; 12 (43%) patients had > 4-weeks’ support (longest was 292 days). Eight (29%) patients died on support. They reported similar complications to our study, including bleeding in 10 (36%) cases, immediate stroke in 4 (14%), and dialysis in 8 (29%) [9]. There was no stroke during subsequent support. Two (7%) patients recovered and were discharged. Two-year survival was 62% ± 10%. The mean follow-up was 21 months (total follow-up 579 months). Two (7%) patients died during follow-up. All of their survivors were reported to be in New York Heart Association class I.

Another study by Zeriouh et al. [10] of 66 patients who had CentriMag support for critical cardiogenic shock showed that the mean duration of support in the survivor group was 35 ± 25 days versus 25 ± 25 days in the non-survivor (n.s.) group, ranged from 1 to 109 days. Their overall survival on support was 40 (60%) patients, which is similar to our survival to successful explant from CentriMag™ of 65%. In the survivor group, 12 patients could be successfully weaned from the system, and 12 patients received a heart transplant and in 16 a long-term VAD was implanted [10].

### Early conversion from ECMO to BiVAD

We convert our patients, within a week, from ECMO to BiVAD as long-term support with ECMO causes more complications. A study by Kurihara et al. [11], studying patients bridged to durable LVAD who were supported with short-term MCS, also showed that survival across all four time points (30 days to 2 years) was poorest for patients supported with VA ECMO (*p* = 0.02).

### Review of complications

Our study showed bleeding requiring intervention occurring in 38% patients. This is partly due to acquired von Willebrand factor deficiency [12]. In vitro study by Coghill et al. showed von willebrand factor (VWF) collagen-binding activity (VWF:CB)/VWF antigen ratio in the HeartMate 2, CentriMag, and HVAD exhibited average decreases of 46%, 44%, and 36% from baseline after 360 min of operation [12]. This damage to platelet function is much higher at higher pump speed. Chen et al. showed that the level of platelet activation, loss of VWF, and loss of key platelet adhesion receptors (GPVI and GPIbα) leading to platelet dysfunction increase with increasing pump speed (rpm) of the CentriMag [13]. Therefore, measures to improve cardiac filling status should be used before increasing pump speed to increase cardiac output.

### Exit strategy

In the initial days, when the recovery of cardiac function remains a possibility, left ventricular distention should be avoided on ECMO, by maintaining sinus rhythm (and promptly shocking out of ventricular fibrillation (VF)), ensuring aortic valve opening on trans-oesophageal echo, or use of LV vent/Impella, or conversion to BiVAD. If heart function does not recover, heart transplantation remains the best option for these patients and should be done as soon as they have recovered from multi-organ failure and are ready to withstand the operation. If heart function does not recover and a suitable heart is not available for transplantation, early crossover to a durable LVAD should be considered as it leads to better overall survival [14].

### Limitations

There are several limitations of this study. First, it was a retrospective analysis of our centre experience. Second, because of the nature of single-centre studies, the outcomes described here are based on our practice in terms of patient selection, surgery, and management. Therefore, our findings may not be directly applicable to other centres.

## Conclusion

Our results demonstrate an excellent outcome can be achieved with the use of the CentriMag™ device for mechanical circulatory support in critical cardiogenic shock patients. Despite requiring multiple procedures, over 58% of patients were successfully discharged from hospital and 5-year survival was 46%.
